# Gas Plasma Exposure Attenuates the Inflammatory Rheumatoid Arthritis‐Like Phenotype of Murine Synoviocytes in Vitro

**DOI:** 10.1111/jcmm.71118

**Published:** 2026-03-26

**Authors:** Marcel Kordt, Wendy Bergmann‐Ewert, Johann Aleith, Sander Bekeschus, Brigitte Müller‐Hilke

**Affiliations:** ^1^ Core Facility for Cell Sorting and Cell Analysis Rostock University Medical Center Rostock Germany; ^2^ Department of Dermatology, Venereology, and Allergology Rostock University Medical Center Rostock Germany; ^3^ ZIK Plasmatis Leibniz Institute for Plasma Science and Technology (INP) Greifswald Germany

**Keywords:** fibroblast‐like synoviocytes, kINPen, plasma medicine, reactive oxygen species, rheumathoid arthritis

## Abstract

Rheumatoid arthritis (RA) is driven by hyperplastic fibroblast‐like synoviocytes (FLS) that adopt a persistent inflammatory and invasive phenotype, often resistant to conventional therapies. Here, we investigated whether cold gas plasma exposure modulates inflammatory FLS (iFLS) in vitro. Primary murine FLS were primed with TNF‐α to induce an inflammatory phenotype and exposed to an argon plasma jet for 30–150 s; argon gas alone served as a control. Gas plasma treatment caused a rapid, dose‐dependent rise in intracellular reactive oxygen species and free thiols, followed by partial resolution by 24 h. This oxidative burst coincided with mitochondrial dysfunction and progressive loss of cell viability. Surviving iFLS exhibited markedly reduced inflammatory features, such as surface levels of ICAM‐1 (CD54), VCAM‐1 (CD106), and Thy‐1 (CD90.2), along with diminished production of IL‐6 and CCL2 at higher doses. Reflecting the impaired expression of inflammation‐associated adhesion molecules. Furthermore, plasma treatment significantly delayed iFLS migration. Together, these findings suggest that cold gas plasma has cytotoxic effects on a subset of iFLS induced by acute oxidative stress, as well as reprogramming surviving iFLS towards a less aggressive phenotype. These in vitro data establish a foundation for further research in synovial explants and arthritis models, as well as for systematic safety testing in non‐inflammatory joint‐resident cells.

## 1 Introduction

Rheumatoid arthritis (RA) is a chronic autoimmune disease characterized by persistent synovial inflammation and progressive joint destruction [1, 2]. In RA, the physiologically thin synovial lining expands into an invasive pannus as immune cells infiltrate the joint, leading to cartilage degradation and bone erosion if left uncontrolled [3]. A central player in pathogenesis is the fibroblast‐like synoviocyte (FLS), which exhibits an aggressive, “tumour‐like” phenotype [4, 5]. RA‐FLS proliferate abnormally and secrete an array of pro‐inflammatory cytokines and matrix‐degrading enzymes, thereby perpetuating chronic inflammation and contributing directly to joint damage [6]. Indeed, RA‐FLS produce mediators such as interleukin‐6 (IL‐6) and IL‐8, chemokines such as C–C motif chemokine ligand 2 (CCL2) and matrix metalloproteinases (MMPs), including MMP1 and MMP3, that drive synovial inflammation and tissue destruction. Moreover, they express receptor activator of nuclear factor κB ligand (RANKL), thereby facilitating osteoclast‐mediated bone resorption [7, 8]. This aberrant, hyperplastic behaviour of RA‐FLS, which is often likened to that of malignant cells, drives pannus formation. This is a process where the synovial lining thickens and invades cartilage and bone tissue [9, 10, 11]. First‐line treatments, like nonsteroidal anti‐inflammatory drugs (NSAIDs), low‐dose glucocorticoids, and conventional synthetic disease‐modifying antirheumatic drugs (csDMARDs), can relieve symptoms and slow disease progression. However, some patients require more advanced biological, targeted, or biosimilar DMARDs, which can increase the risk of infection. Additionally, these treatments do not achieve remission in all patients [1, 12, 13, 14, 15].

Among the myriad inflammatory signals in the RA joint, tumour necrosis factor‐alpha (TNF‐α) plays a pivotal role [16]. In vitro, exposure of FLS to TNF‐α recapitulates many features of the RA inflammatory milieu. TNF‐α stimulation drives FLS to an activated inflammatory phenotype, markedly upregulating the production of IL‐6 and IL‐8 [17]. Exposure of murine FLS to TNF‐α has been shown to induce long‐term transcriptomic and epigenetic reprogramming, effectively ’imprinting’ these cells with a sustained pro‐inflammatory memory [18]. We therefore term these cells ‘inflammatory FLS’ (iFLS). This TNF‐α‐induced iFLS model mirrors the key features of FLS activation in RA joints. It therefore provides a robust platform for evaluating anti‐inflammatory interventions.

Cold gas plasma has emerged over the past decades as a versatile biomedical tool, with dielectric barrier discharge (DBD) and plasma jets among the most widely used clinical devices [19, 20]. Gas plasma is generated by applying an external energy source, typically an electromagnetic field, to a neutral gas. This produces a mixture that is partially ionized, containing electrons, ions, and neutral particles [21, 22]. The resulting plasma contains reactive oxygen and nitrogen species (RONS), along with UV and visible photons, which confer strong chemical reactivity without excessive heat [19, 23]. In oncology research, gas plasma treatment has demonstrated significant anti‐tumor effects in vitro across diverse cancer cell lines and in vivo in subcutaneous xenograft models [23, 24, 25].

Previous studies have primarily examined the effects of gas plasma exposure on FLS viability, apoptosis, and overall inflammatory marker levels [26, 27, 28]. However, the direct impact of gas plasma treatment on cytokine production and inflammatory signalling in proinflammatory FLS remains unclear. This study addresses this gap by focusing on the anti‐inflammatory capacity of gas plasma in iFLS.

## 2 Methods

### 2.1 Cell Isolation and Culture

Primary FLS were isolated from the front and hind paws of 8–10‐week‐old female B10.Q mice according to an established in‐house protocol [29]. In brief, mice were sacrificed under ketamine/xylazine anaesthesia by cervical dislocation, and paws were disinfected with 70% ethanol before dissection. After removal of skin and muscle tissue, joint tissue was enzymatically digested in FLS‐DMEM containing 240 U/mL collagenase type IV (STEMCELL Technologies, Vancouver, Canada) for 1.5 h at 37°C under gentle agitation (400 × rpm). Cells were filtered through a 100 μm cell strainer, pelleted by centrifugation (400 × rpm, 10 min), and cultured in T75 flasks in Dulbecco's Modified Eagle Medium (DMEM; PAN‐Biotech, Aidenbach, Germany) supplemented with 10% fetal bovine serum (FBS) (PAN‐Biotech), 100 U/mL penicillin, and streptomycin (Sigma‐Aldrich, Taufkirchen, Germany). Medium was changed after 3–4 days, and cells were maintained for 7–10 days to reduce macrophage contamination. Cells were preserved at 37°C and 5% CO_2_.

From passage four onwards, FLS were cultured in complete Roswell Park Memorial Institute medium (RPMI 1640; PAN‐Biotech) supplemented with 10% FBS (PAN‐Biotech) and 1% penicillin/streptomycin (Sigma‐Aldrich), due to its lower content of scavengers interfering with ROS‐based assays [30]. Cells were stimulated with 5 ng/mL recombinant mouse TNF‐α (BioLegend, San Diego, CA, USA) for 72 h to induce a proinflammatory phenotype. For plasma treatment, iFLS at passage 5 were seeded at a density of 76,842 cells/cm^2^ in 24‐well plates. After 24 h, the medium was replaced with fresh RPMI containing 5 ng/mL TNF‐α, followed by immediate plasma treatment as described below.

To measure intracellular cytokine expression, the iFLS were incubated in the presence of 5 μg/mL Brefeldin A (BioLegend) at 37 C for 5 h before harvesting.

### 2.2 Cold Gas Plasma Device and Treatment

The cold gas plasma device was supplied by the Leibniz Institute for Plasma Science and Technology (INP, Greifswald, Germany). For treatment, the high‐frequency plasma jet kINPen (neoplas, Greifswald, Germany) was used. The kINPen belongs to a well‐characterized non‐thermal atmospheric‐pressure plasma jet family that has been extensively described with regard to its physical and chemical properties, plasma diagnostics, and medical application context. Its characterization has also contributed to the development of DIN SPEC 91315 for medical plasma sources [31]. It operates at temperatures between 35°C and 50°C [32]. The plasma jet contains a grounded outer electrode and operates at a frequency of 1 MHz with a pin‐type powered electrode inside a 1.6 mm‐thick dielectric ceramic tube. The kINPen has a dissipated power of 3.5 W and uses a direct current power supply with a maximum system energy of 50 W at 100–240 V, 50/60 Hz. The jet was operated using argon as the feed gas at a flow rate of 1.5 standard litres per minute (slm). The operating distance from the pencil to the medium surface of the 24‐well plate was ∼7 mm. Using a computer‐controlled and motorized XYZ table (CNC, Bremen, Germany), the kINPen hovered over the center of each well. Furthermore, a control condition (CTRL) was implemented, wherein cells were exposed to the feed gas argon alone, without ionization, in order to account for any effects on gas flow pressure. Non‐stimulated FLS without plasma served as baseline controls.

### 2.3 Flow Cytometry

#### 2.3.1 Initial Assessment of Gas Plasma‐Induced Cytotoxicity

After 24‐h gas plasma exposure, iFLS were harvested, washed twice with phosphate‐buffered saline (PBS), and incubated for 30 min at room temperature (RT) in dark with a staining mix containing MitoSpy Orange CMTMRos (500 nM; BioLegend) and Zombie NIR Fixable Viability Dye (1:1000; BioLegend) in PBS. Cells were centrifuged (400 × g, 5 min, RT), resuspended in autoMACS Running Buffer (RB, Miltenyi Biotec, Germany, Bergisch Gladbach), and acquired on a BD FACSVerse cytometer (Becton Dickinson (BD) Biosciences, Franklin Lakes, NJ).

#### 2.3.2 Evaluation of Gas Plasma‐Induced Oxidative Stress

For intracellular ROS measurements, culture supernatants were aspirated 2, 4, or 24 h after gas plasma treatment, and wells were washed twice with PBS to remove extracellular ROS. iFLS were then incubated in situ with 5 μM CellROX Green (Thermo Fisher Scientific) for 30 min at 37 C. Subsequently, cells were trypsinised (0.25% Trypsin/EDTA; PAN‐Biotech) for 3 min, neutralized with medium, and washed twice with PBS. Cells were resuspended in 100 μL PBS containing Zombie NIR (1:1000), ThiolTracker Violet (0.625 μM; Thermo Fisher Scientific), and MitoSpy Orange (500 nM; BioLegend) and incubated for 30 min at RT in the dark. Following two washes with RB, samples were analysed on a Cytek Aurora spectral flow cytometer (Cytek Biosciences, Fremont, CA) operated using SpectroFlo Software v3.2.1.

#### 2.3.3 Phenotypic and Cytokine Profiling

For the comprehensive phenotyping and intracellular cytokine analysis, iFLS were stained in a final volume of 100 μL as described. After two PBS washes, cells were incubated for 30 min at RT in the dark with Zombie NIR (1:1000) and MitoSpy Orange (500 nM). After centrifugating and washing with RB, cells were fixed using 1× Fixation Buffer (BioLegend) for 20 min at RT, permeabilized with 1× Intracellular Staining Perm Wash Buffer (BioLegend), and subsequently incubated with 10% FCS, 1 μg anti‐CD16/32 (clone 93), and 5% True Monocyte Blocker (BioLegend) blocking solution for 15 min. Subsequently, all cells were stained intracellularly for 20 min at RT in the dark (Table 1).

**TABLE 1:** Antibody panel for intracellular cytokine/chemokine phenotyping. Per‐test amounts refer to 100 μL staining volume.

Target	Clone	Fluorochrome	Amount (μg)	Vendor
MHC II	M5/114.152	PE‐Fire 640	0.12	BioLegend
CD54	YN1/1.74	PerCP‐Cy5.5	0.125	BioLegend
CD31	390	SuperBright 436	0.25	Thermo Fisher
CD45	30‐F11	APC‐Fire 810	1.0	BioLegend
TNF‐α	MP6‐XT22	BV421	1.0	BioLegend
CD11b	M1/70	AF660	0.5	Thermo Fisher
CD254/RANKL	REA1026	PE‐Vio 615	0.075	Miltenyi Biotec
CD86	PO3.3	APC‐Vio 770	0.075	Miltenyi Biotec
CD106	429	BV480	0.5	BD Biosciences
CCL2	2H5	PE	0.5	BioLegend
F4/80	T45‐2342	R718	0.5	BD Biosciences
CD90.2	30‐H12	VioBlue	0.0375	Miltenyi Biotec
CD80	16‐10A1	BV605	1.0	BioLegend
CCL3	REA355	APC	0.075	Miltenyi Biotec
IL‐6	REA1034	Vio667	0.06	Miltenyi Biotec

After staining, cells were washed twice with 1× Intracellular Staining Perm Wash Buffer and once with RB, resuspended in 500 μL RB, and analysed using a CytekAurora. Data were analysed using FlowJo (v10.9; FlowJo, Ashland, OR, USA).

### 2.4 LEGENDplex Multiplex Cytokine Assay

Cell culture supernatants were collected 24 h after gas plasma treatment and left undiluted. Cytokine concentrations were determined using the LEGENDplex Mouse Inflammation Panel (BioLegend) according to the manufacturer's instructions. The following analytes were included: IFN‐γ, TNF‐α, MCP‐1/CCL2, IL‐1β, IL‐10, IL‐6, and IL‐17A. Briefly, 25 μL of standards or samples were incubated with 25 μL of mixed capture beads and 25 μL of detection antibodies for 2 h at RT on a plate shaker (800 rpm), followed by the addition of 25 μL streptavidin‐PE for 30 min. After each incubation, plates were washed once with the supplied wash buffer and centrifuged. Beads were resuspended in 200 μL assay buffer and acquired on a CytekAurora. FCS files were uploaded to the LEGENDplex Data Analysis Software (cloud‐based). Concentrations were calculated from standard curves using a five‐parameter logistic (5‐PL) fit.

### 2.5 Scratch (Migration) Assay

After 24 h of seeding 1.46 × 10^5^ iFLS cells per well in 24‐well plates, a linear scratch was created through the centre of each monolayer using a sterile 100 μL pipette tip. Wells were gently washed twice with sterile PBS to remove detached cells and debris. Fresh RPMI supplemented with 10% FCS, 1% penicillin/streptomycin, and 5 ng/mL recombinant mouse TNF‐α was then added, and cells were exposed to gas plasma. Images of the scratch area were captured at 1, 24, and 48 h post‐treatment using a Zeiss Axio Vert. A1 FL‐LED inverted microscope equipped with an Axiocam 305 mono camera (Carl Zeiss Microscopy, Jena, Germany). Distance between cell edges per scratch was measured using ImageJ software (v1.54g; National Institutes of Health, Bethesda, MD, USA). Percent wound closure was calculated by comparing the scratch area at each time point to the 1 h baseline.

### 2.6 Statistical Analysis

Data visualization and statistical analyses were performed using Prism (v8.0.2; GraphPad Software, La Jolla, CA, USA), and some data visualizations were performed using RStudio (v 2025.05.0 (496); Posit Software, PBC, Boston, MA, USA). Values were expressed as mean ± standard deviation (SD) or as median from minimum to maximum for the indicated number of samples. The Shapiro–Wilk normality test was employed to assess the Gaussian distribution. To ascertain significant differences between groups, the Kruskal–Wallis test was conducted for data that did not follow a Gaussian distribution. Two‐way ANOVA with the Geisser–Greenhouse correction and the Dunnett post hoc test was performed to evaluate changes between argon control (CTRL) and treatment duration or initial time point and later time points. The *p*‐values of ≤ 0.05 were considered statistically significant. Significance levels are abbreviated as follows: *p ≤ 0.05, **p ≤ 0.01, ***p ≤ 0.001, ****p ≤ 0.0001.

## 3 Results

### 3.1 Gas Plasma Exposure Triggers Sustained Oxidative Stress and Progressive Loss of Viability in Inflammatory FLS

Before interrogating gas plasma effects, we optimized three critical parameters. Firstly, titration of TNF‐α (0–10 ng/mL) revealed that 5 ng/mL robustly increased ICAM‐1 (CD54), VCAM‐1 (CD106), and MHC II (I‐A/I‐E) on FLS, while higher doses yielded only marginal additional activation but significantly lower cell yields. Thus, 5 ng/mL was chosen for all subsequent experiments (Supplementary Figure 1A). Secondly, based on the lower total Aantioxidant capacity of RPMI compared to DMEM [30], we tested whether RPMI would enhance gas plasma‐mediated cell toxicity. In a representative iFLS sample, RPMI was found to yield stronger gas plasma‐induced effects and permit shorter treatment times. We therefore performed all gas plasma experiments in RPMI to maximize oxidative stress (Supplementary Figure 1B). Thirdly, we knew that cell density can influence the apparent gas plasma‐induced toxicity. This means that a higher effective ROS dose per cell is achieved with fewer cells per well [32, 33, 34]. Thus, we varied the seeding densities. Extremely low densities resulted in near‐complete cell death due to the high RONS dose per cell, even at moderate gas plasma exposure time (60 s) (Supplementary Figure 1C). We settled on 146,000 cells/well as the density that forms a near confluent monolayer. This avoided overestimation of the gas plasma‐induced lethality. These optimizations ensured a robust iFLS phenotype and reproducible gas plasma effects for subsequent analyses.

**FIGURE 1:**
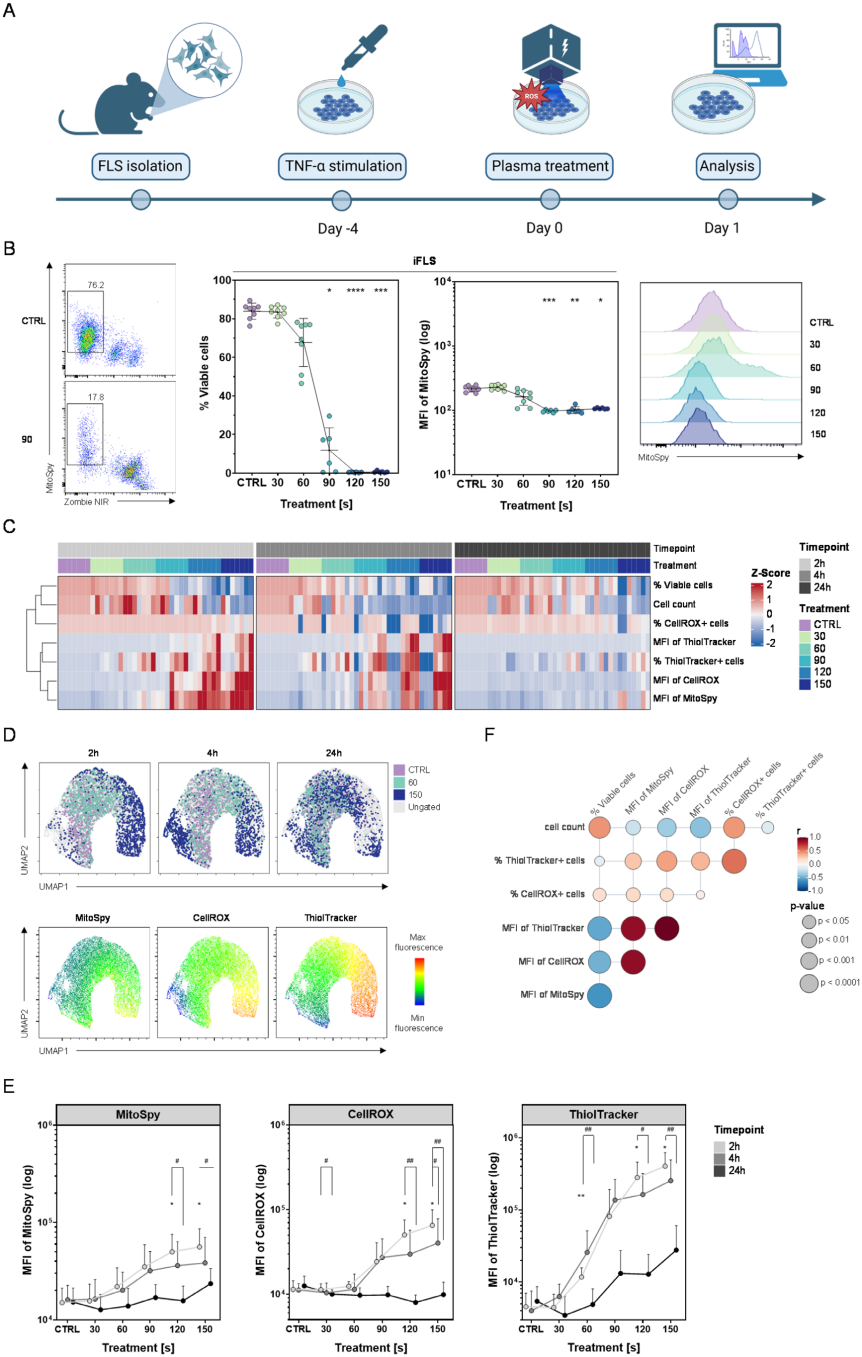
Cold gas plasma exposure induced time‐ and dose‐dependent oxidative and viability changes in inflammatory FLS (iFLS). (A) Schematic of the experimental workflow: IFLS were seeded in 24‐well plates, stimulated with 5 ng/mL TNF‐α, and treated with gas plasma for varying durations. (B) Representative flow‐cytometric measurements of cell viability and mitochondrial membrane potential 24 h after gas plasma treatment. Quantitative analysis of viability and cell count (*n* = 8, two independent experiments). (C) Heatmap of Z‐score–normalized mitochondrial potential (MitoSpy), intracellular ROS (CellROX Green), and thiol content (ThiolTracker Violet) at 2, 4, and 24 h after gas plasma treatment; data clustered by treatment and timepoint. (D) Uniform manifold approximation and projection (UMAP) embedding of single‐cell fluorescence profiles (MitoSpy, CellROX, ThiolTracker) across all timepoints and treatments (showed CTRL, 60 s, and 150 s), illustrating distinct separation of gas plasma‐exposed populations. (E) Quantitative analysis of CellROX, ThiolTracker, and MitoSpy at 2, 4, and 24 h after gas plasma treatment. (F) Bubble‐plot correlation matrix of all measured parameters showing the strength and direction of associations across treatments and timepoints. C to E *n* = 7; two independent experiments; Kruskal–Wallis with the Dunn's post hoc test; *p < 0.05, **p < 0.01, ***p < 0.001, ****p < 0.0001.

As outlined in the experimental schematic (Figure 1A), upon exposing iFLS to cold plasma, we first evaluated cell viability and mitochondrial health 24 h post‐treatment (Figure 1B). Compared to the argon control group, gas plasma treatment durations ranging from 90 to 150 s resulted in a significant decrease (*p* < 0.05 for 90 s and *p* < 0.001 for 150 s) in the live‐cell fraction and a reduction in mitochondrial membrane potential (approximately 50% less MitoSpy fluorescence signal). This suggests that prolonged oxidative stress damages mitochondrial integrity and reduces survival. Shorter exposures (30 or 60 s) had more modest effects, with cell viability similar to the control group after 24 h. However, a trend of decreased MitoSpy intensity was evident even after 60 s. To address potential off‐target toxicity, we performed a donor‐matched viability titration in unstimulated FLS and compared the results with those of TNF‐α–conditioned iFLS (Supplementary Figure 1D). Plasma reduced viability in both conditions at higher doses. However, the responses were heterogeneous between donor pairs and not statistically significant. To delineate the temporal dynamics of this response, we measured redox markers at 2, 4, and 24 h (Figure 1C). Plasma treatment leads to an early increase in intracellular ROS concentrations (MFI CellROX) and, as a consequence of redox homeostasis and adaptation, an increase in free thiol (MFI ThiolTracker) as early as 2 h after plasma intervention, with peak values at very long treatment durations. This trend continued after 4 h, with a slight decrease. By 24 h, ROS levels partially decrease but remain slightly elevated compared to controls, whereas the surviving cells ramp up their antioxidant capacity. Meanwhile, an increase in mitochondrial potential occurred very early (after 2 h), indicating hyperpolarization of the mitochondrial membrane as a result of the initial oxidative stress.

As shown in Figure 1D, control cells cluster distinctly from those exposed to 150 s gas plasma at 2 h and 4 h, demonstrating that plasma generates a unique, dose‐dependent phenotypic signature. Whereas the 60 s treatment group clusters closer to cells from the control. Overlaying MitoSpy, CellROX, and ThiolTracker intensities onto the UMAP projection visualizes how individual cells traverse from a low‐stress to a high‐stress state in response to gas plasma. As shown in Figure 1E, higher durations of gas plasma (120 s and 150 s) result in a significant mitochondrial hyperpolarization and an increase in ROS and thiol markers (*p* < 0.05 to *p* < 0.01 compared to argon control), particularly 2 h and 4 h after gas plasma treatment. Following a 24‐h period, there was a decline in mitochondrial membrane potential and intracellular ROS levels. However, the thiol content remained relatively high in the surviving cells. Correlating all these parameters (Figure 1F), it becomes obvious that gas plasma induced an early increase in intracellular ROS levels, which is accompanied by mitochondrial hyperpolarization and a significant enhancement of the cellular antioxidant capacity. Together, these data demonstrate that gas plasma exposure provokes an acute and persistent oxidative challenge in iFLS, undermining mitochondrial function and viability.

### 3.2 Gas Plasma Treatment Dampens the Expression of Adhesion Molecules and Pro‐Inflammatory Cytokines

**FIGURE 2:**
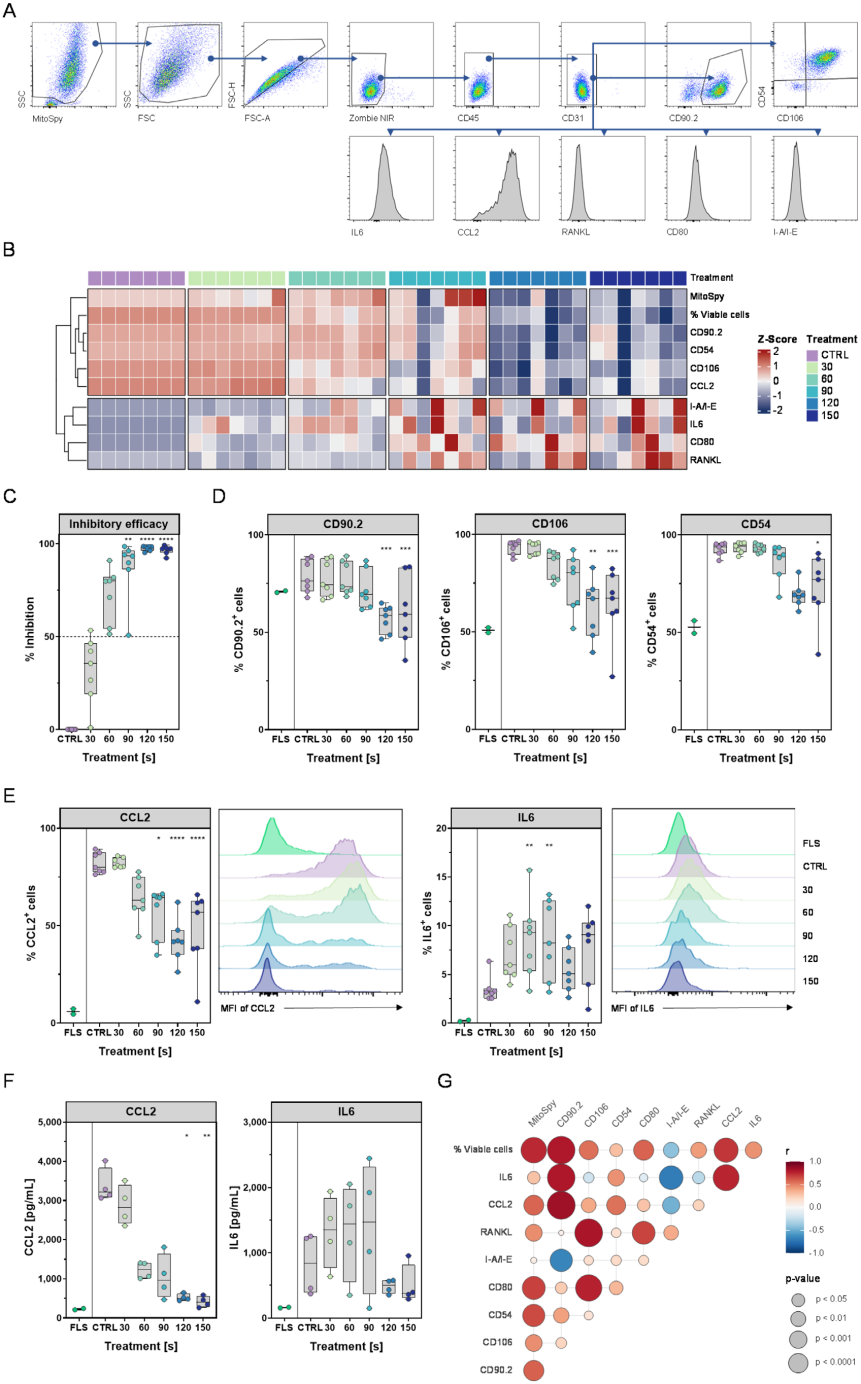
Cold gas plasma treatment dampened inflammatory marker expression and cytokine release in inflammatory FLS (iFLS). (A) Gating strategy shown on the argon control (CTRL) 24 h after gas plasma treatment. (B) Heatmap of Z‐score–normalized median fluorescence intensities (MFIs) for CD90.2 (Thy‐1), CD106 (VCAM‐1), CD54 (ICAM‐1), CD80, RANKL (CD254), I‐A/I‐E (MHC II), intracellular CCL2, and IL‐6, plus % viable cells across gas plasma treatment (CTRL, 30–150 s). (C) Inhibitory efficacy (% inhibition vs. CTRL) calculated from the % viable cells and cell count. (D and E) Percentages of marker‐positive cells (CD90.2, CD106, CD54, CCL2, and IL‐6) 24 h after gas plasma treatment. FLS were characterized as TNF‐α and gas plasma‐treated and untreated cells. (F) Secreted CCL2 and IL‐6 (pg/mL) quantified by LEGENDplex 24 h after treatment. (G) Bubble‐plot correlation matrix of all measured parameters showing the strength and direction of associations across treatments. (C to E and G) *n* = 7; two independent experiments; (F) *n* = 4; Kruskal–Wallis with the Dunn's post hoc test; *p < 0.05, **p < 0.01, ***p < 0.001, ****p < 0.0001.

Next, we investigated the impact of plasma exposure on the inflammatory phenotype of the surviving iFLS. For these experiments, iFLS were treated with gas plasma and analysed 24 h later for surface markers and cytokine production (gating strategy is illustrated in Figure 2A). Live FLS were gated as CD45^−^, CD31^−^, and ZombieNIR^−^ cells (Figure 2A). We then assessed markers of FLS activation (ICAM‐1/CD54, VCAM‐1/CD106, Thy‐1/CD90.2, and CD80), antigen presentation (MHC II), inflammatory mediators (intracellular IL‐6, CCL2), and pro‐osteoclastogenic marker (Receptor Activator of NF‐κB Ligand (RANKL)).

Figure 2B provides an overview of the modulation of the markers using gas plasma treatment. It is striking that a concerted downward shift in the expression of several key molecules can be observed with increasing gas plasma duration. The surface levels (MFI) of CD90.2, CD106, and CD54 continued to decrease as the duration of gas plasma treatment increased. The chemokine CCL2 also showed a dose‐dependent decrease as a result of plasma treatment. In contrast, the MFI of I‐A/I‐E, which was low at the beginning of treatment, was slightly elevated with medium gas plasma exposure and even more markedly with 150 s gas plasma. However, this was not observed in all samples. IL‐6 signals also tended to increase with prolonged gas plasma treatment duration. In line with these observations, the MFI of RANKL and the co‐stimulatory molecule CD80 also rose. Together, this is reminiscent of the surviving cells due to accelerated apoptosis. In agreement with previous results, the percentage of live cells was simultaneously reduced at ≥ 90 s gas plasma treatment (Figure 2B). This broad pattern suggests that gas plasma induces oxidative stress (as shown in Figure 1) and reprograms the inflammatory phenotype of iFLS by reducing their adhesion markers and chemokine production. To better quantify these effects, we calculated an “inhibitory efficacy” metric for each gas plasma dose (Figure 2C). This metric summarizes the gas plasma's overall suppressive impact by integrating the reduction in total live cell numbers relative to the control. It already shows a trend towards an increase in inhibition at 30 and 60 s treatment time, with maximum inhibition occurring at higher durations. Next, we examined specific marker‐positive fractions in surviving cells. Figure 2D shows the percentage of iFLS positive for CD90.2, CD106, and CD54 after gas plasma treatment. Between 85 and 95% of the argon‐control iFLS were positive for ICAM‐1 and VCAM‐1, reflecting the fully activated state. Compared to non‐stimulated FLS, only around 53% of cells expressed CD54 and 51% expressed CD106. Gas plasma treatment caused a significant decline in the expression of these three markers. For instance, the proportion of CD90.2^+^ cells decreased from a median of 76.4% in the control group to 69.4% after 90 s of plasma treatment and to 59.2% after 150 s (*p* < 0.001). Thus, the expression of CD90.2 decreased to below that of non‐TNF‐α‐stimulated FLS (~71%). The effect was more pronounced for adhesion molecules: CD54^+^ cells declined from ~94% (control) to ~77% at 150 s gas plasma treatment (*p* < 0.05). CD106^+^ cells fell from ~95% (control) to ~67% at 150 s gas plasma exposure (*p* < 0.001). These reductions are functionally relevant, indicating diminished adhesion and activation potential. Given that VCAM‐1 and ICAM‐1 on FLS facilitate leukocyte retention and synovial infiltration in RA [9], a lower level of these markers could suggest reduced joint inflammation.

We also assessed the expression of inflammatory cytokines in iFLS following plasma exposure. Figure 2E shows the percentage of cells positive for intracellular CCL2 and IL‐6 under each condition. Only around 6% of non‐stimulated FLS expressed CCL2. In contrast, in TNF‐α‐activated controls, the vast majority of iFLS (~80%) expressed CCL2. Following gas plasma treatment, there was a significant decline in CCL2^+^ cells. After 90 s of gas plasma treatment, only ~64% of cells were CCL2^+^ (*p* < 0.05). This decline continued at 150 s, with only ~57% of cells remaining positive (a slight recovery compared to 120 s, but still significantly below the argon control, *p* < 0.0001). This suggests that gas plasma treatment can suppress CCL2 expression at the cellular level, beyond the reduction expected from cell death alone. In contrast, IL‐6 was produced by a smaller subset of iFLS (~3% of cells in control). IL‐6^+^ cells exhibited a non‐monotonic pattern. At 30 to 90 s of gas plasma treatment, the IL‐6^+^ fraction was slightly higher than the control group (60 s ~9%, 90 s ~8%, *p* < 0.01). This suggests that moderate stress does not immediately reduce IL‐6 and may even stimulate transient IL‐6 production in some cells. However, at 120 s, IL‐6^+^ cells drop to ~5% (comparable to control). Nevertheless, when considering total IL‐6 output, the combination of fewer IL‐6^+^ cells and fewer total cells implies a net reduction in IL‐6 production by the cell population.

To validate this hypothesis and the intracellular staining results, we measured the concentrations of the secreted cytokines CCL2 and IL‐6 in the supernatant after 24 h (Figure 2F). In the control group, iFLS cells secreted high levels of CCL2 (~3200 pg/mL) and IL‐6 (~840 pg/mL) after 24 h, but plasma treatment decreased these levels. Secreted CCL2 was reduced to ~1200 pg/mL after 60s gas plasma treatment and further to ~500–400 pg/mL after 120 (*p* < 0.05 vs. control) to 150 s gas plasma exposure (*p* < 0.01 vs. control). IL‐6 secretion exhibited a comparable trend to that observed in the intracellular staining analysis. IL‐6 concentration increased moderately after just 30 s of gas plasma treatment (~61%). However, it was only with longer treatment intervals of 120 and 150 s that IL‐6 concentrations were reduced to ~500 and 380 pg/mL, respectively, which was slightly below the control values. These data confirm that gas plasma treatment attenuates the inflammatory response of iFLS, causing them to release much lower concentrations of IL‐6 and CCL2 into their environment. This is consistent with the immunomodulatory properties of plasma reported in other contexts [26, 35]. CCL2 is a key chemokine that recruits monocytes to inflamed joints, while IL‐6 is a major driver of systemic and local inflammation. Both are elevated in RA‐FLS and are clinically targeted, for example, through IL‐6 blockade [1, 36].

To explore relationships between markers, we performed correlation analysis on the multi‐parameter flow data (Figure 2G). This revealed that the surface marker CD90.2 was strongly positively correlated with intracellular IL‐6 and CCL2 expression across individual cells. Conversely, I‐A/I‐E expression showed an inverse correlation with IL‐6, CCL2, and CD90.2. This suggests that within our iFLS cultures, cells inclined towards cytokine production (IL‐6^+^/CCL2^+^) were distinct from any cells that upregulated I‐A/I‐E. Notably, I‐A/I‐E remained very low on all conditions (only ~0.5% positive in argon controls, rising to at most ~2.5% at 150 s gas plasma exposure; see Figure S2).

Importantly, we verified that the gas plasma‐induced phenotypic changes we observed at 24 h were largely maintained through 48 h post‐treatment (Figure S2A). Across almost all gas plasma treatment durations, total iFLS counts increased between 24 and 48 h, reflecting expected cell proliferation during this interval, except following only 30 s of gas plasma exposure, where cell recovery remained limited (Figure S2B). Similarly, the percentage of IL‐6^+^ cells declined at 48 h for all treatments except 120 s, although the MFI of IL‐6 staining was unchanged, indicating stable per‐cell cytokine content among the remaining positive cells. We also noted minor fluctuations in the MFI of CCL2, CD54, CD90.2, I‐A/I‐E, and MitoSpy between 24 and 48 h under certain gas plasma conditions. These data confirm that gas plasma's immunomodulatory and cytotoxic effects on iFLS are both rapidly elicited and durable over at least 48 h. Together, these findings demonstrate that gas plasma treatment not only compromises iFLS viability (Figure 1) but also actively down‐modulates their inflammatory and adhesive phenotype.

### 3.3 Gas Plasma‐Mediated Suppression of Adhesion Molecules Attenuates FLS Migration

**FIGURE 3:**
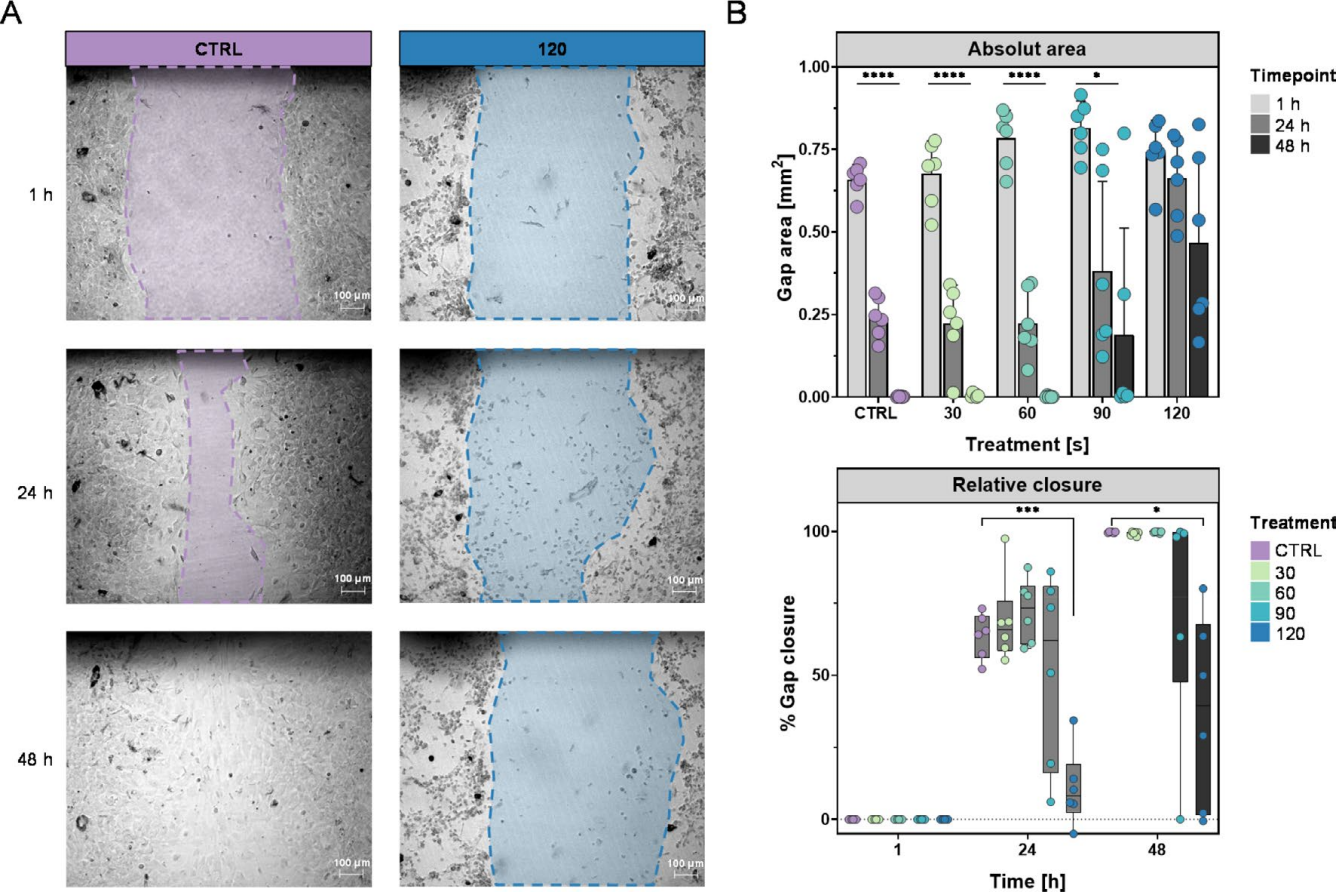
Cold gas plasma exposure impaired scratch closure of inflammatory FLS (iFLS) in a treatment time‐dependent manner. (A) Representative phase‐contrast images of the scratch wound in iFLS monolayers (scale bar = 100 μm) for argon control (CTRL) and 120 s gas plasma treatment at 1, 24, and 48 h after treatment. (B) Quantification of absolute gap area (mm^2^) across gas plasma doses (CTRL, 30–120 s) and relative gap closure (%) over time (1, 24, and 48 h). Data are shown as mean ± SD for absolute area and median from minimum to maximum for relative closure; *n* = 6 total (three biological replicates × two technical replicates). Two‐way ANOVA with the Geisser–Greenhouse correction and Dunnett post hoc test; *p < 0.05, ***p < 0.001, ****p < 0.0001.

One hallmark of activated RA‐FLS is their ability for migration and tissue invasion, partly through the influence of ICAM‐1 and VCAM‐1. Blocking of these molecules markedly reduces migratory and invasive capacity [37, 38, 39]. Since gas plasma treatment reduces adhesion molecule expression (Figures 2B and 2D), we asked whether this translates into functional changes in cell motility. Therefore, we performed a scratch assay where iFLS were exposed to gas plasma, and gap closure was monitored over 48 h (Figures 3A and S3A). One hour post‐scratch, the initial gap area was similar across all conditions at around 0.8 mm^2^ (Figure 3B). After 24 h, iFLS treated with argon alone had migrated into the gap, achieving approximately 60% closure (Figure 3C). Strikingly, iFLS that received a 120 s plasma treatment failed to repopulate the area effectively. The gap remained largely open until 48 h, with only minimal cell migration from the edges. This confirmed that gas plasma exposure impaired the migration capacity of iFLS in a dose‐dependent manner. Interestingly, we noted a modestly accelerated closure at 24 h compared with the control at lower gas plasma doses, suggesting a potential eustress effect of short gas plasma exposure on migration. Similar biphasic responses have been reported in fibroblasts, where sublethal gas plasma treatment has been shown to enhance scratch‐wound closure, likely via ROS‐mediated activation [40, 41]. Together, these data show that gas plasma‐mediated suppression of ICAM‐1/VCAM‐1 on iFLS not only alters their inflammatory phenotype (Figure 2), but also impairs their migratory capacity, which is an essential contributor to pannus formation and joint invasion in RA.

## 4 Discussion

RA is characterized by a hyperplastic, inflamed synovium teeming with activated FLS and infiltrating immune cells [1, 4]. These RA‐FLS exhibit an autonomous, aggressive phenotype that not only sustains inflammation through cytokine and chemokine production but also directly drives joint destruction through tissue invasion and matrix degradation [3, 14]. Targeting the pathogenic behaviour of RA‐FLS is increasingly recognized as a therapeutic strategy for RA [6, 42]. In this study, we explored the use of cold plasma as a novel tool for modulating inflammatory FLS in an in vitro model of RA. Our findings demonstrate that gas plasma treatment can attenuate key pathological features of TNF‐α‐conditioned FLS in vitro: it induced (i) oxidative stress leading to (ii) apoptosis in iFLS, (iii) down‐regulated inflammatory signalling (IL‐6, CCL2), (iv) adhesion molecules (VCAM‐1, ICAM‐1), and (v) impaired migration capacity. These effects suggest gas plasma exposure could help “reset” the synovial microenvironment from a pro‐inflammatory, tissue‐invasive state towards a more resolved state.

Gas plasma exposure rapidly elevated intracellular ROS and induced thiol increase as a sign of antioxidative defence within hours. This is consistent with the known capacity of plasma‐generated RONS to penetrate cells and disrupt redox balance [21, 43]. Similar redox perturbations have been linked to activation of stress kinases, like p38 mitogen‐activated protein kinase (MAPK), c‐Jun N‐terminal kinase (JNK) [44], and mitochondrial permeability transition, leading to depolarization and apoptotic priming [45, 46]. The transient nature of ROS elevation in our experiments, peaking at 2–4 h and resolving partially by 24 h, suggests the activation of endogenous antioxidant responses, possibly via nuclear factor erythroid 2‐related factor 2 (NRF2) [47]. One potential hypothesis is that sublethal gas plasma doses trigger a beneficial stress response in iFLS, whereby short‐term oxidative stress enhances specific repair or migration pathways, as has been observed in dermal fibroblasts [40].

We observed a dose‐dependent decline in viable iFLS. This aligns with previous reports on cancer and fibroblast models, in which plasma‐induced oxidative damage exceeds the cellular antioxidant capacity, resulting in apoptosis or necrosis [26, 27]. Interestingly, residual populations persisted even at high doses, which may reflect the intrinsic heterogeneity in the redox sensitivity of iFLS subpopulations undergoing reprogramming [18, 48]. Gas plasma treatment significantly reduced expression of VCAM‐1 and ICAM‐1, as well as the FLS marker Thy‐1, which is known for its high inflammatory nature in CD90^+^ RA fibroblasts [49]. These two adhesion molecules mediate leukocyte recruitment [50, 51] and synovial retention, but also contribute to motility [37, 38, 39]. Thus, their suppression may directly attenuate both immune cell–FLS interactions and FLS autonomous movement. Given that TNF‐α strongly induces both molecules [52], the findings suggest that plasma interferes with downstream transcriptional or post‐transcriptional regulation of TNF‐responsive genes, potentially through redox inhibition of transcription factors such as NF‐κB [26, 53].

Following gas plasma treatment, the proportion of iFLS producing CCL2 and its secretion into the supernatant, decreased. The same was true for IL‐6, albeit to a lesser extent. Both of these cytokines play a central role in the pathology of RA. IL‐6 drives synovial inflammation and systemic symptoms, while CCL2 recruits monocytes to the joint [1, 6]. Redox modulation has been shown to influence cytokine gene expression by altering transcription factor DNA binding [54]. A reasonable hypothesis is that gas plasma‐induced oxidative stress transiently inhibits NF‐κB pathways [53], thereby reducing the transcription of pro‐inflammatory genes. This reduction in pro‐inflammatory mediators is supported by prior plasma studies in RA‐FLS [26] and M1 macrophages [55], which also leads to reduced infiltration of inflammatory macrophages.

At higher doses, plasma exposure led to reduced migration and thus to delayed closure of the iFLS gap. This is probably due not only to reduced cell viability but also to diminished adhesion molecule expression. However, at sublethal exposures (30 and 60 s), we observed slightly accelerated closure at 24 h, reminiscent of a beneficial migration‐promoting effect [40, 56]. This biphasic response may result from ROS‐mediated activation of focal adhesion turnover and cytoskeletal remodelling at low doses, versus cytoskeletal collapse and detachment at high doses [57, 58]. Importantly, FLS migration is a critical contributor to pannus expansion and joint invasion [1] The suppression is a plausible mechanism by which gas plasma treatment could attenuate disease progression if similar effects occur in vivo.

Looking ahead, non‐invasive manipulation of the synovial microenvironment to increase the transdermal flow of longer‐lasting reactive species could be interesting [59, 60]. Preclinical cold plasma delivery in arthritis models has ranged from the direct treatment of surgically exposed synovium to minimally invasive, joint‐cavity‐directed plasma devices [27, 28]. It is important to note that we do not envision an “open‐joint” strategy. Instead, any future in vivo work should prioritize clinically realistic, non‐ or minimally invasive delivery concepts. Barrier modulation could be combined with topical formulations to prolong residence time at the surface, or with thin, flexible, dielectric barrier “plasma patches” that maintain low dosage at the skin surface without damaging tissue [60, 61]. For deeper penetration, microneedle‐based patches would be a suitable option. These patches have already been demonstrated for transdermal immunotherapy with cold plasma.

## 5 Conclusion

Our data suggest that gas plasma treatment could be used alongside other treatments for RA, targeting FLS through two complementary mechanisms: direct cytotoxicity towards hyperactivated subsets and functional reprogramming of survivors towards a less invasive, less inflammatory phenotype. Although these observations were made in vitro, they provide a mechanistic framework for further exploration in synovial explants and/or in vivo arthritis models. Key questions that need answering include how to optimize cytotoxic and modulatory plasma effects, how to ensure sufficient tissue penetration, and what the potential off‐target effects on other synovial cell types are.

## Author Contributions

M.K. performed the experiments, B.M.‐H., M.K., and S.B. designed and conducted the research, M.K. analysedanalyzed the data, M.K. wrote the manuscriptpaper, and B.M.‐H., J.A., M.K., S.B., and W.B.‐E. also edited the manuscript. All authors contributed to manuscript revision, and read and approved the submitted version.

## Consent

The authors have nothing to report.

## Conflicts of Interest

The authors declare no conflicts of interest.

## Supporting information


**Figure S1:** Optimisation of TNF‐α dose, culture medium, and seeding density for iFLS before cold gas pressure plasma experiments. A TNF‐α titration (0, 1, 5, and 10 ng/mL) to induce an inflammatory phenotype. Flow cytometry 24 h after stimulation (*n* = 3). B Medium comparison (single biological replicate): iFLS stimulated with 5 ng/mL TNFα were cultured in either DMEM (standard FLS medium) or RPMI. C Seeding density test (36,500–292,000 cells/well) to account for cell‐density–dependent ROS toxicity. All measurements were performed by flow cytometry 24 h after gas plasma treatment (*n* = 4). D Comparison of gas plasma‐induced cytotoxicity in unstimulated FLS and TNF‐α stimulated iFLS. Cell viability was assessed by flow cytometry 24 h after treatment (CTRL, 90 s, 120 s, and 150 s). Data are shown as paired donor samples (*n* = 3). A to C One‐way ANOVA with Dunnett's post hoc test; D paired two tailed *t*‐Test; two independent experiments; *p < 0.05, **p < 0.01, ***p < 0.001, ****p < 0.0001.
**Figure S2:** Persistence of cold gas plasma‐induced phenotypic modulation in iFLS at 24 h versus 48 h after treatment. Flow cytometric measurement of iFLS 24 and 48 h after gas plasma treatment. A Heatmap of Z‐score–normalized median fluorescence intensities (MFIs) and marker‐positive cell frequencies for surface and intracellular markers. Data are shown for all gas plasma exposure durations (CTRL, 30, 60, 90, 120, and 150 s). B Direct comparison of each parameter at 24 h versus 48 h for each gas plasma duration (*n* = 7, two independent experiments). Wilcoxon matched‐pairs test; *p < 0.05, **p < 0.01, ***p < 0.001.
**Figure S3:** Complete representative image series of scratch closure in inflammatory FLS (iFLS) after cold gas plasma treatment. A representative phase‐contrast images of scratch wounds in iFLS monolayers (scale bar = 100 μm) following Argon CTRL, 30 s, 60 s, 90 s, and 120 s gas plasma treatment, recorded 1 h, 24 h, and 48 h after treatment. To enable direct visual comparison across the full‐dose range, the complete image series from one representative mouse is shown, including the control and 120 s conditions presented in Figure 3.

## Data Availability

The data that support the findings of this study are available from the corresponding author upon reasonable request.
