# Supplementary material for: Gas Plasma Exposure Attenuates the Inflammatory Rheumatoid Arthritis‐Like Phenotype of Murine Synoviocytes in Vitro
Source: J Cell Mol Med. 2026 Mar 26;30(6):e71118. doi: 10.1111/jcmm.71118 (PMC13097525; doi:10.1111/jcmm.71118)
Supplement: Supplementary file 1 — Figure S1: Optimisation of TNF‐α dose, culture medium, and seeding density for iFLS before cold gas pressure plasma experiments. A TNF‐α titration (0, 1, 5, and 10 ng/mL) to induce an inflammatory phenotype. Flow cytometry 24 h after stimulation (n = 3). B Medium comparison (single biological replicate): iFLS stimulated with 5 ng/mL TNFα were cultured in either DMEM (standard FLS medium) or RPMI. C Seeding density test (36,500–292,000 cells/well) to account for cell‐density–dependent ROS toxicity. All measurements were performed by flow cytometry 24 h after gas plasma treatment (n = 4). D Comparison of gas plasma‐induced cytotoxicity in unstimulated FLS and TNF‐α stimulated iFLS. Cell viability was assessed by flow cytometry 24 h after treatment (CTRL, 90 s, 120 s, and 150 s). Data are shown as paired donor samples (n = 3). A to C One‐way ANOVA with Dunnett's post hoc test; D paired two tailed t‐Test; two independent experiments; *p < 0.05, **p < 0.01, ***p < 0.001, ****p < 0.0001. Figure S2: Persistence of cold gas plasma‐induced phenotypic modulation in iFLS at 24 h versus 48 h after treatment. Flow cytometric measurement of iFLS 24 and 48 h after gas plasma treatment. A Heatmap of Z‐score–normalized median fluorescence intensities (MFIs) and marker‐positive cell frequencies for surface and intracellular markers. Data are shown for all gas plasma exposure durations (CTRL, 30, 60, 90, 120, and 150 s). B Direct comparison of each parameter at 24 h versus 48 h for each gas plasma duration (n = 7, two independent experiments). Wilcoxon matched‐pairs test; *p < 0.05, **p < 0.01, ***p < 0.001. Figure S3: Complete representative image series of scratch closure in inflammatory FLS (iFLS) after cold gas plasma treatment. A representative phase‐contrast images of scratch wounds in iFLS monolayers (scale bar = 100 μm) following Argon CTRL, 30 s, 60 s, 90 s, and 120 s gas plasma treatment, recorded 1 h, 24 h, and 48 h after treatment. To enable direct visual comparison across [file JCMM-30-e71118-s001.docx]

**Figure S1. Optimization of TNF-α dose, culture medium, and seeding density for iFLS prior to cold gas pressure plasma experiments. A** TNF-α titration (0, 1, 5, 10 ng/mL) to induce an inflammatory phenotype. Flow cytometry 24 h after stimulation (n = 3). **B** Medium comparison (single biological replicate): iFLS stimulated with 5 ng/mL TNFα were cultured in either DMEM (standard FLS medium) or RPMI. **C** Seeding density test (36,500–292,000 cells/well) to account for cell‑density–dependent ROS toxicity. All measurements were performed by flow cytometry 24 h after gas plasma treatment (n = 4). **D** Comparison of gas plasma-induced cytotoxicity in unstimulated FLS and TNF-α stimulated iFLS. Cell viability was assessed by flow cytometry 24 h after treatment (CTRL, 90 s, 120 s, 150 s). Data are shown as paired donor samples (n = 3). **A** to **C** One‑way ANOVA with Dunnett's post hoc test; **D** paired two tailed t-Test; two independent experiments; *p < 0.05, **p < 0.01, ***p < 0.001, ****p < 0.0001.

**Figure S2. Persistence of cold gas plasma-induced phenotypic modulation in iFLS at 24 h versus 48 h after treatment.** Flow cytometric measurement of iFLS 24 and 48 hours after gas plasma treatment. **A** Heatmap of Z‑score–normalized median fluorescence intensities (MFIs) and marker‑positive cell frequencies for surface and intracellular markers. Data are shown for all gas plasma exposure durations (CTRL, 30, 60, 90, 120, 150 s). **B** Direct comparison of each parameter at 24 h versus 48 h for each gas plasma duration (n = 7, two independent experiments). Wilcoxon matched‑pairs test; *p < 0.05, **p < 0.01, ***p < 0.001.

**Figure S3. Complete representative image series of scratch closure in inflammatory FLS (iFLS) after cold gas plasma treatment. A** Representative phase-contrast images of scratch wounds in iFLS monolayers (**scale bar = 100 µm**) following **Argon CTRL, 30 s, 60 s, 90 s, and 120 s** gas plasma treatment, recorded **1 h, 24 h, and 48 h** after treatment. To enable direct visual comparison across the full dose range, the complete image series from **one representative mouse** is shown, including the **control and 120 s** conditions presented in Figure 3.
